# Are survival outcomes dependent on the tumour dose threshold of 139 Gy in patients with chemorefractory metastatic colorectal cancer treated with yttrium-90 radioembolization using glass particles? A real-world single-centre study

**DOI:** 10.1093/bjr/tqae096

**Published:** 2024-05-10

**Authors:** Osman Melih Topcuoglu, Tolga Orhan, Ayşegul Gormez, Nalan Alan

**Affiliations:** Department of Radiology, Yeditepe University Hospitals, Kosuyolu 34718, Turkey; Department of Radiology, Yeditepe University Hospitals, Kosuyolu 34718, Turkey; Department of Radiology, Yeditepe University Hospitals, Kosuyolu 34718, Turkey; Department of Nuclear Medicine, Yeditepe University Hospitals, Kosuyolu 34718, Turkey

**Keywords:** colorectal liver metastases, radioembolization, TARE, 90Y

## Abstract

**Objectives:**

To compare the survival and objective response rate (ORR) of the patients receiving estimated tumour absorbed dose (ETAD) <140 Gy versus ETAD ≥140 Gy in patients with advanced chemorefractory colorectal carcinoma liver metastases (CRCLM) treated with yttrium-90 transarterial radioembolization (90Y TARE).

**Methods:**

Between August 2016 and August 2023 adult patients with unresectable, chemorefractory CRCLM treated with 90Y TARE using glass particles, were retrospectively enrolled. Primary outcomes were overall survival (OS) and hepatic progression free survival (hPFS). Secondary outcome was ORR.

**Results:**

A total of 40 patients with a mean age of 66.2 ± 7.8 years met the inclusion criteria. Mean ETAD for group 1 (ETAD <140 Gy) and group 2 (ETAD ≥140) were 131.2 ± 17.4 Gy versus 195 ± 45.6 Gy, respectively. The mean OS and hPFS for group 1 versus group 2 were 12 ± 10.3 months and 8.1 ± 9.3 months versus 9.3 ± 3 months and 7.1 ± 8.4 months, respectively and there were no significant differences (*P* = .181 and *P* = .366, respectively). ORR did not show significant difference between the groups (*P* = .432).

**Conclusion:**

In real-world practice, no significant difference was found in OS, hPFS, and ORR between patients who received ETAD <140 Gy versus ETAD ≥140 Gy in patients with CRCLM, in this series.

**Advances in knowledge:**

This study demonstrated that increased tumour absorbed doses in radioembolization may not provide additional significant advantage for OS and hPFS for patients with CRCLM.

## Introduction

In recent years, studies investigating the dose-response relationship in the treatment of metastatic and primary liver malignancies with transarterial radioembolization (TARE) have provided important new information. Improvements and changes in the concept of dosimetry are among the latest innovations necessary to perform a safe and effective selective internal radiotherapy using yttrium-90 (90Y)-loaded microspheres.^1^^,^^2^ Dosimetry in TARE depends on the amount of absorbed dose from ionising radiation. A macro-aggregated albumin (MAA) scan can be obtained prior to TARE to assess the estimated tumour absorbed dose (ETAD), or direct 90Y distribution can be used after treatment.^1^^,^^3^ However, the latter does not allow to use personalized dosimetry. The term “personalised dosimetry”, which has been recommended in several recent publications, means adjusting the amount and location of the administered activity based on each patient's hepatic arterial anatomy, specific tumour absorbed dose, and extrahepatic shunting.^2^^,^^4^^,^^5–13^ In addition, increased ETAD has been shown to be significantly associated with improved tumour response and becomes a critical component of TARE.^5–7^ Threshold dose levels for tumour tissue death and normal liver parenchymal tolerance have also been studied separately for glass and resin particles, in primary and metastatic liver tumours.^1^^,^^5–7^ Alsultan et al^5^ reported a minimum threshold of 139 Gy for tumouricidal effect and 189 Gy for better overall survival (OS) in patients with colorectal cancer liver metastases (CRCLM) that were treated with glass microspheres. The effect of 90Y doses below and above 139 Gy on OS remains to be determined in CRCLM treated with Therasphere particles.

The aim of the current study was to compare the survival and objective response rate (ORR) of patients receiving ETAD <140 Gy versus ETAD ≥140 Gy in patients with advanced chemorefractory CRCLM treated with 90Y TARE using glass particles.

## Methods

### Patient population

The local ethics committee approved this single-centre retrospective study, and the requirement for obtaining informed consent was waived. The study harboured chemorefractory adult patients with unresectable CRCLM treated with 90Y TARE using Therasphere particles between August 2016 and August 2023.

**Figure 1.**
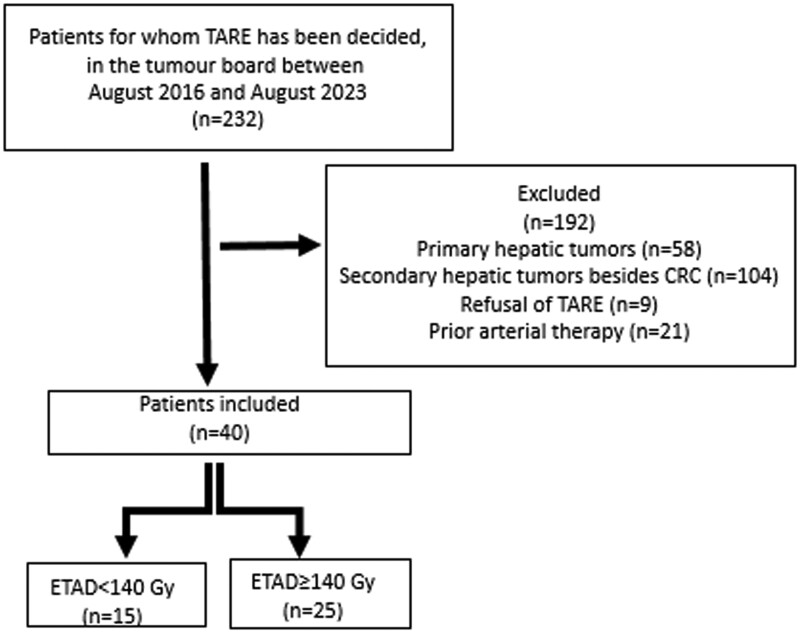
Study flowchart. Abbreviations: TARE = transarterial radioembolization; ETAD = estimated tumour absorbed dose.

All patients had received first- and second-line chemotherapy and showed progressive disease. Number of metastases, lobar involvement (unilobar versus bilobar), origin of the CRC (right-sided versus left-sided), presence of portal vein thrombosis, and extrahepatic disease were noted. Age <18 years, bilirubin >1.2 upper normal limit, albumin ≤2.8 g/dL, prior arterial therapy as chemoembolization or radiotherapy to the liver, clinically evident ascites, unresolved hepatic toxicity from first- or second-line therapy, lung shunt greater than 30 Gy, resectable disease, and contraindication to angiography were the exclusion criteria. All treatments were decided in a local multi-disciplinary tumour board. Study flowchart is given in Figure 1.

### Procedure

All procedures were performed *via* standard right femoral approach under local anaesthesia. A reverse-angle 5F catheter (USL-2 or Sim1) was used for coeliac trunk catheterization. Microcatheters with a size of 2.8F (Progreat, Terumo, Somerset, New Jersey, USA, or Embocath, BioSphere Medical, Rockland, MA) were used for the deemed hepatic artery selective catheterization. Technetium-99m (99mTc)-MAA infusion was performed from the placed microcatheter either from the right or the left hepatic artery. A decision to proceed with 90Y treatment was made depending on the single photon emission CT (SPECT-CT) findings. 90Y-labeled microspheres (Therasphere™; Boston Scientific, Marlborough, MA, United States) were delivered from the identical catheter localization. Lobar or sequential bilobar treatment was performed. No antibiotic prophylaxis was used. All procedures were performed by an interventional radiologist with more than 10 years of individual experience in embolization procedures.

### Definitions and outcomes

Primary outcomes were OS and hepatic progression free survival (hPFS). Secondary outcome was ORR. Imaging was performed using MRI or fluorodeoxy glucose PET (FDG PET) at baseline and every 8 weeks thereafter. ORR (complete response—CR, partial response—PR, stable disease—SD, and progressive disease—PD) was determined according to response evaluation criteria in solid tumours 1.1 (RECIST 1.1) or positron emission tomography response criteria in solid tumours (PERCIST). Two radiologists, one with two years' experience and one with more than 10 years' experience in liver imaging, evaluated the pre- and post-procedure images blinded to each other. Response assessment results were then compared and the agreement was evaluated by intraclass correlation coefficients (ICC). In case of discrepancies, a decision was made through a separate consensus meeting. Respond to 90Y TARE was defined as CR + PR, whereas non-respond was defined as SD + PD. Outcomes were also assessed for patients with right-sided and left-sided CRC. The observed adverse events were assessed following National Cancer Institute's Common Terminology Criteria for Adverse Events (CTCAE v5.0).

### Dose calculation

Dosimetry planning was made using a multi-compartment model with respect to the medical internal radiation dose (MIRD) approach. Depiction of tumours and image assessments were performed on Simplicit90Y^®^ software (Mirada Medical Ltd.). Personalized dosimetry was utilized for treatment planning in all patients as per standard of care. ETAD was calculated from the 99mTc-MAA scans.

### Statistical analysis

The study population was divided into two groups as patients receiving ETAD of <140 Gy versus those of ≥140 Gy. Categorical variables were given as percentages, and continuous variables were reported as means and standard variations. The differences between the groups were assessed using the chi-square test and Mann-Whitney *U* test. Variables with a probability value <.10 in the univariate analysis and with clinical relevance were selected for the models. The confidence interval was 95%. Inter-reader agreement was assessed with ICC. Kaplan-Meier analysis was used for OS and hPFS. OS was accepted as the time between radioembolization and death from any reason. *P* < .05 was considered as significant. All analyses were performed with SPSS 25.0 (Statistical Package for the Social Sciences).

## Results

A total of 40 patients (26 men and 14 women) with a mean age of 64.4 ± 9.6 years met the inclusion criteria. Thirty-nine patients had bilobar disease. Eleven patients had right-sided and 29 patients had left-sided primary colon carcinoma. Total number of metastases were ≤6 (*n* = 6), 6-10 (*n* = 8), and >10 (*n* = 26). There was no detected portal vein thrombosis or vascular invasion. Group 1 and group 2 included 15 and 25 patients, respectively. Extrahepatic disease was present in 53.3% of the patients in group 1 and 68% of the patients in group 2 and there was no significant difference (*P* = .502) (Table 1). Mean ETAD for group 1 (ETAD <140 Gy) and group 2 (ETAD ≥140) was 131.2 ± 17.4 Gy versus 195 ± 45.6 Gy, respectively.

**Table 1.** Association between estimated tumour absorbed doses and study parameters.

Parameter	ETAD <140 Gy (*n* = 15)	ETAD ≥140 Gy (*n* = 25)	*P*-value
Mean ETAD	132.2 ± 16.9 Gy	196.9 ± 45.5 Gy	.500
Extrahepatic disease	8 (53.3%)	17 (68%)	.502
ECOG status			.168
0	10 (66.6%)	18 (72%)	
1	4 (26.6 %)	6 (24%)	
≥2	1 (6.6%)	1 (4%)	
Tumour distribution			.923
Unilobar	–	1 (4%)	
Bilobar	15 (100%)	24 (96%)	
Hepatic tumour burden %			.072
<10	4 (26.6%)	7 (28%)	
≥10 to <25	3 (20%)	5 (20%)	
≥25	8 (53.3%)	13 (52%)	
Overall survival	12 ± 10.3 months	9.3 ± 3 months	.181
hPFS	8.1 ± 9.3 months	7.1 ± 8.4 months	.366

Abbreviations: ETAD = estimated tumour absorbed dose; ECOG = eastern cooperative oncology group; hPFS = hepatic progression free survival.

**Figure 2.**
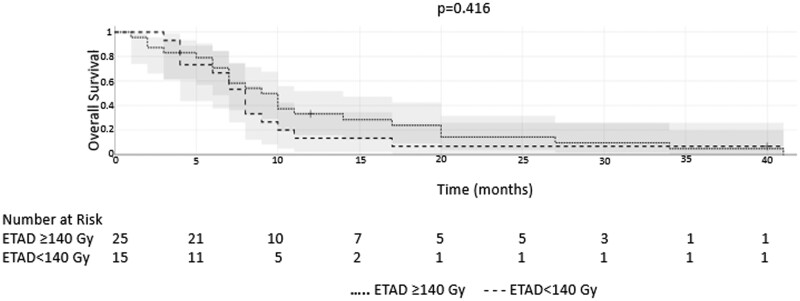
Kaplan-Meier analysis of overall survival for patients receiving ETAD ≥140 Gy versus ETAD <140 Gy. Abbreviation: ETAD = estimated tumour absorbed dose.

**Figure 3.**
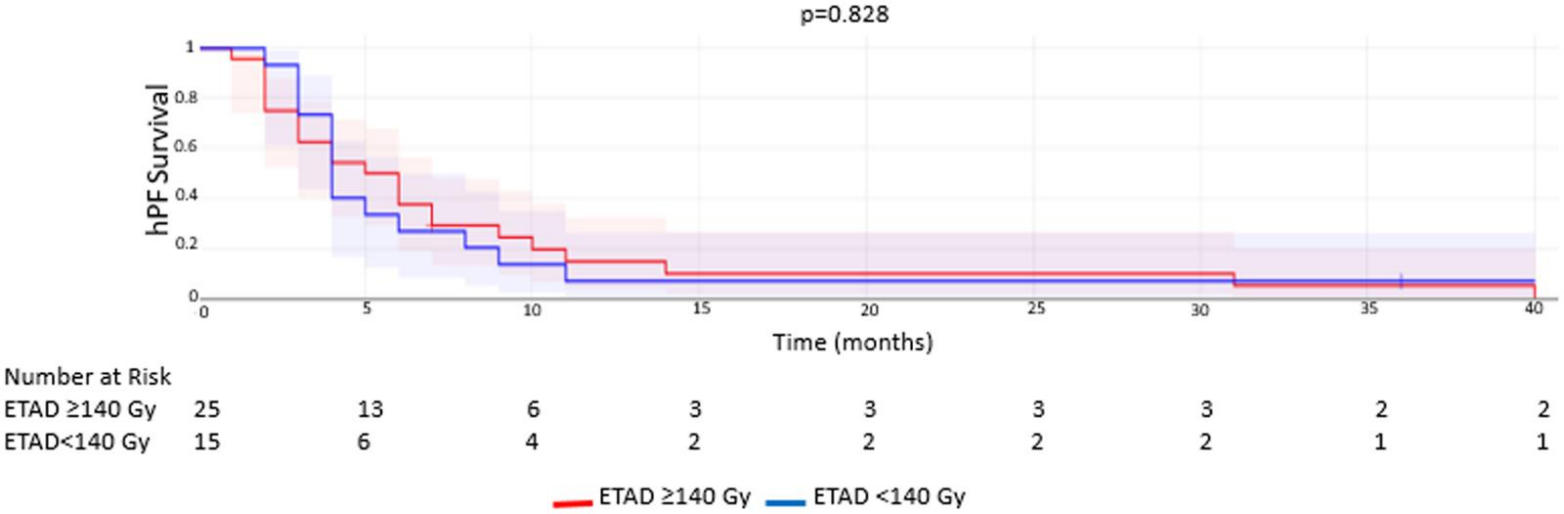
Kaplan-Meier analysis of hepatic progression free survival for patients receiving ETAD ≥140 Gy versus ETAD <140 Gy. Abbreviations: ETAD = estimated tumour absorbed dose; hPF = hepatic progression free.

The mean OS and hPFS in the whole sample were 11.1 ± 9.6 months (median 8 months) and 8.4 ± 4.5 months (median 6 months), respectively. The mean OS and hPFS for group 1 versus group 2 were 12 ± 10.3 and 8.1 ± 9.3 months versus 9.3 ± 3 and 7.1 ± 8.4 months, respectively and there were no significant differences (*P* = .181 and *P* = .366, respectively) (Table 1). Kaplan-Meier analyses are shown in Figures 2 and 3. Patients with right-sided primary tumours showed less OS after 90Y RE compared to patients with left-sided primary tumours (6.1 ± 3 months versus 12.1 ± 10 months; respectively, *P* = .052).

ORR did not show significant difference between the groups (*P* = .432). ORR with respect to the ETAD is summarized in Table 2. The mean ETAD for responders (CR + PR, *n* = 27) and non-responders (SD + PD, *n* = 13) was 171.3 ± 46.3 Gy and 155.7 ± 34.5 Gy, respectively. The mean ETAD in responders was only 10.3% higher than in patients with PD and this difference was not significant (*P* = .184). The mean OS for responders (CR + PR) and non-responders (SD + PD) was 9.3 ± 10 months and 4 ± 2.7 months, respectively (*P* = .052). Patients showing complete response at the first imaging follow-up (*n* = 8) had significantly better OS than patients showing PR or PD or SD (16.8 ± 12.3 months versus 9.4 ± 7.9 months, respectively) (*P* = .022). Inter-reader agreement was excellent with an ICC value of 0.934 (95% confidence intervals between 0.881 and 0.967).

**Table 2.** Objective response rates with respect to the estimated tumour absorbed doses.

Outcome	ETAD <140 Gy (*n* = 15)	ETAD ≥140 Gy (*n* = 25)	*P*-value
Overall response, no. (%)			
CR	2 (13.3%)	6 (24%)	.414
PR	7 (46.6%)	12 (48%)	.934
SD	1 (6.6%)	–	–
PD	5 (33.3%)	6 (24%)	.522
NA	–	1 (4%)	–
Objective response rate	9 (60%)	18 (72%)	.432

The mean ETAD for patients with right-sided primary tumours versus left-sided primary tumours was 169.4 ± 58.8 Gy versus 171.9 ± 43.1 Gy (*P* = .454). Responders in patients with right-sided primary tumours did not show significant difference compared to responders in patients with left-sided primary tumours (50% versus 71.8%, respectively, *P* = .237).

Grade 3 or 4 adverse events were not detected in both the groups.

## Discussion

It has been found that there was no significant difference between patients receiving ETAD <140 Gy versus ETAD ≥140 Gy, in terms of OS, hPFS, and ORR in patients with CRCLM, in this series. The probability of ORR and OS was shown to be higher with increasing tumour absorbed dose for hepatocellular carcinoma in the TARGET study.^14^ Alsultan et al^5^ reported that the mean ETAD in responders (CR + PR) was 58% higher than in patients with PD in CRCLM, whereas in the current study, only a 10.3% difference in increased tumour absorbed dose was found between responders and patients with PD. They also found that ETAD greater than 139 Gy anticipated ORR with the greatest accuracy and ETAD greater than 189 Gy predicted OS with a specificity of 99%. In contrast, in the current study, there was no significant difference in ORR between patients who received ETAD ≥140 Gy and those who received ETAD less than 140 Gy, at three months. In this series, estimated tumour doses above and below 140 Gy did not show a significant difference in OS and hPFS. There are a number of possible explanations for the differences in results between the studies. First, only 24 patients were included in the dose-response evaluation and the development of new metastases after TARE was not taken into count. The metastases themselves, not the patients, were used for statistical analysis. Finally, their study used the post-treatment 90Y distribution to calculate dose thresholds for tumouricidal activity in patients with CRCLM. In this study, the pre-treatment 99mTc-MAA distribution was used for dose calculation.

A median OS of 8 months was found in this single centre cohort and Helmberger et al^15^ published a median OS of 9.8 months for CRCLM after TARE with resin particles, similar to the results of the current study. The slightly higher OS in their prospective study, with data obtained from 68 different centres across Europe, than the OS results of the current study may be due to the presence of a higher number of patients with extrahepatic disease (62.5%), in this series. As in real-life practice, all patients in the current study had received first- and second-line chemotherapy regimens and when the CRCLM progressed, patients were then treated with Y90 TARE as a salvage therapy. Due to this workflow, the timing of TARE remained late and was used as a rescue or palliative option for patients with CRCLM. OS could be further improved, if the timing of TARE could be brought forward. Furthermore, the prevalence of extrahepatic disease precludes the OS benefit of TARE as a locoregional therapy.

The results of the current study also revealed that OS following TARE in CRCLM was not solely dependent on the ETAD, and that in addition to tumour absorbed doses, the anatomical origin of the primary CRC also influenced OS. Wu et al^16^ found that right-sided primary tumours were independently associated with lower OS compared to the left-sided primary tumours, in patients with CRCLM following 90Y TARE, although all those patients had similar ORR and hPFS. In parallel, we have found significantly lower OS for patients with right-sided primary tumours than those with left-sided primary tumours, despite these patients received similar ETAD. In other words, although these patients with CRCLM receive high tumour absorbed doses during TARE, the original anatomical tumour location and its usual behaviour still determine the natural disease course and OS. Because right-sided CRC has its different molecular and immunological characteristics that determine the routine clinical course, it is more difficult to increase OS by planning higher ETAD compared to left-sided tumours.^17–19^

Mulcahy et al^20^ demonstrated no OS advantage with the addition of glass-based TARE to the second-line systemic chemotherapy in patients with CRCLM. They utilized 120 ± 10 Gy for treatments and in the current study, despite there were although no observed grade 3 or higher adverse events were observed, increased ETAD did not provide additional advantage for OS and hPFS. The latest American Society of Clinical Oncology (ASCO) guideline also does not recommend routine use of TARE in unresectable CRCLM, as no survival benefit has been shown.^21^

There were some limitations in this study. First of all, relatively small sample size and retrospective design were the main limitations. Furthermore, results of the current study need to be approached with caution as this was a retrospective study susceptible to multiple forms of bias with a very small sample size. Secondly, although the ORR assessments were carried out by two radiologists, there was no consistency in the type of imaging modality that was used, such as MRI and FDG PET. Thirdly, the dosimetry was based on the pre-treatment MAA distribution only and the post-treatment 90Y distribution which does not allow for personalized dosimetry, was not used. However, recent papers supported the current study’s dosimetry planning method and revealed that pre-treatment MAA is strongly correlated with the post-90Y PET, in terms of the mean absorbed dose and also the dose distribution.^22^^,^^23^ Fourthly, the administered sphere count as dose/sphere was not calculated for each patient. The availability of flexible dose shipping options may have resulted in different dose/sphere values for different administrations, resulting in varying sphere density and dose uniformity/heterogeneity.^24^^,^^25^ This important point might cause misinterpretation of the actual dose-response relationship. Finally, the current study included chemorefractory CRCLM patients with extensive prior treatment, therefore the results may not reflect the TARE for patients with less prior treatment. Nevertheless, changing dosimetry approaches in TARE towards personalization, necessitates finding out tumouricidal tumour absorbed doses that provide survival benefit while preserving the normal healthy liver tissue.

In conclusion, in real-world practice, no significant difference was found in OS, hPFS, and ORR between patients who received ETAD <140 Gy versus ETAD ≥140 Gy, in patients with CRCLM, in this series.
